# Serological and Molecular Investigation of Batai Virus Infections in Ruminants from the State of Saxony-Anhalt, Germany, 2018

**DOI:** 10.3390/v13030370

**Published:** 2021-02-26

**Authors:** Nicole Cichon, Martin Eiden, Jana Schulz, Anne Günther, Patrick Wysocki, Cora M. Holicki, Joachim Borgwardt, Wolfgang Gaede, Martin H. Groschup, Ute Ziegler

**Affiliations:** 1 Institute of Novel and Emerging Infectious Diseases, Friedrich-Loeffler-Institut, Federal Research Institute for Animal Health, 17493 Greifswald-Insel Riems, Germany; nic.cichon@web.de (N.C.); martin.eiden@fli.de (M.E.); cora.holicki@fli.de (C.M.H.); martin.groschup@fli.de (M.H.G.); 2 Institute of Epidemiology, Friedrich-Loeffler-Institut, Federal Research Institute for Animal Health, 17493 Greifswald-Insel Riems, Germany; jana.schulz@fli.de (J.S.); patrick.wysocki@fli.de (P.W.); 3 Institute of Diagnostic Virology, Friedrich-Loeffler-Institut, Federal Research Institute for Animal Health, 17493 Greifswald-Insel Riems, Germany; anne.guenther@fli.de; 4 Department of Veterinary Medicine, State Office for Consumer Protection of Saxony-Anhalt, 39576 Stendal, Germany; joachim.borgwardt@sachsen-anhalt.de (J.B.); wolfgang.gaede@sachsen-anhalt.de (W.G.)

**Keywords:** Batai virus, ELISA, seroprevalence, ruminants, Germany

## Abstract

Arthropod-borne Batai virus (BATV) is an *Orthobunyavirus* widely distributed throughout European livestock and has, in the past, been linked to febrile diseases in humans. In Germany, BATV was found in mosquitoes and in one captive harbor seal, and antibodies were recently detected in various ruminant species. We have, therefore, conducted a follow-up study in ruminants from Saxony-Anhalt, the most affected region in Eastern Germany. A total of 325 blood samples from apparently healthy sheep, goats, and cattle were tested using a BATV-specific qRT-PCR and SNT. Even though viral RNA was not detected, the presence of antibodies was confirmed in the sera of all three species: sheep (16.5%), goats (18.3%), and cattle (41.4%). Sera were further analyzed by a glycoprotein Gc-based indirect ELISA to evaluate Gc-derived antibodies as a basis for a new serological test for BATV infections. Interestingly, the presence of neutralizing antibodies was not directly linked to the presence of BATV Gc antibodies. Overall, our results illustrate the high frequency of BATV infections in ruminants in Eastern Germany.

## 1. Introduction

Batai virus (BATV) is one of the most widespread members of the genus *Orthobunyavirus* within the family *Peribunyaviridae*. Its distribution ranges from Malaysia to Asian Russia and India, and in Europe, from Scandinavia to Italy and Romania [1]. In Africa, the virus was described as Ilesha virus in Sudan, Cameroon, Nigeria, Uganda, and Central Africa [1,2].

The negative-strand RNA genome is segmented into small, medium, and large (S, M, and L) segments and encodes four structural and two non-structural proteins. The S segment encodes the N and NSs proteins, the M segment encodes the two glycoproteins Gn and Gc and the NSm protein, and the L segment encodes the RNA-dependent RNA polymerase. The glycoproteins and the non-structural NSm protein are encoded as a precursor polyprotein that is cotranslationally processed by host proteases to produce the three proteins [3]. Especially, the glycoprotein Gc is supposed to be responsible for virus attachment and entry into vertebrate and invertebrate cells [3]. Here, we describe the expression and purification of a BATV Gc subunit and the establishment of an indirect ELISA to subsequently screen ruminant sera for Gc-specific antibodies.

As an arthropod-borne virus, BATV is transmitted in a domestic animal–zoophilic mosquito cycle [2]. Vertebrate hosts include pigs, horses, ruminants, and several bird species [2]. Infections with the Chittor strain in India were reported to cause mild diseases in sheep and goats [4]. In contrast, in Europe, BATV-associated disease has not yet been described in ruminants. However, a BATV infection was detected in a German captive harbor seal with manifested encephalitis [5]. In humans, infections with BATV have been associated with influenza-like symptoms including fever, bronchopneumonia, tonsillitis, and gastritis [6]. The main vectors for BATV in Europe are mosquitoes of the species *Anopheles maculipennis* sensu lato, *Anopheles claviger*, *Coquillettidia richiardii*, and, less often, *Ochlerotatus punctor* and *Ochlerotatus communis* [2]. Two molecular surveys in 2009 (in Southwest Germany) [7] and 2012/2013 (in Northeast Germany) [8] revealed the presence of BATV in anopheline and culicine mosquitoes as well as BATV antibodies in ruminants [9]. The present study was implemented as a follow-up study of the previously mentioned surveys. For this purpose, 325 blood samples from sheep, goats, and cattle were collected in total in Saxony-Anhalt in Eastern Germany, selecting flocks that had shown a high prevalence of BATV in an earlier study [9]. Moreover, we evaluated an indirect ELISA for the screening of BATV Gc-specific antibodies in ruminants.

## 2. Materials and Methods

### 2.1. Sample Collection

A total of 325 blood samples (serum and plasma) from apparently healthy sheep, goats, and cattle were provided by the State Office for Consumer Protection of Saxony-Anhalt in Stendal, Germany. In detail, 60 goats from four flocks, 121 sheep from 11 flocks, and 144 cattle from 13 flocks were sampled in 2018, covering the whole state of Saxony-Anhalt and collected from 10th September to 6th November 2018.

### 2.2. Ethics Statement

Blood samples were collected during obligatory monitoring schemes for other diseases by the State Office for Consumer Protection of Saxony-Anhalt, and provided for our study.

### 2.3. Quantitative Reverse Transcription PCR

RNA isolation from serum and plasma was performed using TRIzol LS Reagent (Life Technologies, Carlsbad, CA, USA) and the Viral RNA Mini Kit (Qiagen, Hilden, Germany) following the manufacturers’ instructions. The quantitative reverse transcription polymerase chain reaction (qRT-PCR) for BATV was carried out according to a previously published protocol using primers and probes which target a 99-nucleotide region of the S segment [7]. Additionally, as an internal control system, IC2 was included [10], using a duplex real-time PCR.

### 2.4. Serum Neutralization Test

All the serum samples from the sheep, goats, and cattle were analyzed in a virus-specific serum neutralization test (SNT), using BATV strain 53.2 (Accession Number HQ455790, kindly provided by J. Schmidt-Chanasit, Bernhard Nocht Institute for Tropical Medicine (BNITM), Hamburg, Germany). The SNT was performed as described by Seidowski et al. [11] and Ziegler et al. [12]. Minor modifications were made by using Vero E6 cells (Collection of Cell Lines in Veterinary Medicine, Friedrich-Loeffler-Institute (FLI), Germany) and applying an incubation time of 6 days. Briefly, a virus concentration of 100 tissue culture infective doses per well (TCID_50_/well) was added to each sample running in duplicate at a starting serum dilution of 1:10. Cytopathic effects were seen 4–6 days post infection. The neutralizing antibody titer of the samples was defined as the 50% neutralization dose (ND_50_). ND_50_ results of 10 or higher were considered positive.

### 2.5. Recombinant Glycoprotein Gc

A synthetic gene optimized for the expression in *E. coli* was produced by Eurofins based on a partial BATV sequence (Accession Number HQ455791) encompassing nucleotide positions 601–1650. The sequence code for the putative domains I and II of the glycoprotein Gc was cloned into the E. coli expression vector pET21a using 5′ BamHI and 3′ XhoI restrictions sites and expressed in BL21-Lys cells. Expression of the recombinant protein and purification by nickel-chelating agarose was carried out under denaturing conditions as described before [13]. Finally, the protein was dialyzed against 0.05 M carbonate–bicarbonate buffer pH 9.6 and checked by SDS-PAGE and Coomassie staining.

### 2.6. Indirect ELISA

The novel indirect in-house ELISA is based on a partial recombinant BATV glycoprotein Gc which was used for coating immunoplates at a dilution of 2 µg/mL in 0.05 M carbonate–bicarbonate buffer pH 9.6 (100 µL per well). Protocol parameters, dilutions, optimal reagent concentrations, and the selection of immunoplates were determined by standard checkerboard titration and the combination with the highest positive to negative control difference (in optical density (OD) values) was chosen. After overnight incubation of the coated immunoplates at 4 °C, plates were washed three times with 300 µL washing buffer containing phosphate-buffered saline (PBS) pH 7.2 and 0.1% Tween 20. After blocking with 200 µL/well 10% skim milk powder (DIFCO™, Thermo Fisher Scientific, Schwerte, Germany) diluted in PBS for 1 h at 37 °C in a moist chamber, ruminant field sera in a dilution of 1:10 in PBS containing 2% skim milk were added in duplicate to the plates. As positive controls, polyclonal hyperimmune rabbit and sheep sera were diluted 1:20 and 1:10, respectively. One hundred-microliter sera dilutions and controls were added to the plates. After incubation at 37 °C for 1 h in a moist chamber, plates were again washed three times with washing buffer. One hundred microliters per well of horseradish peroxidase (HRPO)-conjugated Protein G (Calbiochem^®^, Merck KGaA, Darmstadt, Germany) diluted 1:5000 in dilution buffer were then added to the wells and incubated again for 1h as described before. After a final washing step, 100 µL per well of 2,2-azinodiethylbenzothiazoline sulfonic acid (ABTS, Roche, Mannheim, Germany) substrate were added and plates were incubated for 30 min at room temperature in the dark. The reaction was stopped by adding 1% sodium dodecyl sulfate (SDS) and the OD values were determined at 405 nm. The results were expressed as a percentage of the positive control serum (PP value) using the following formula: (mean OD of duplicate test serum/median OD of duplicate positive control) * 100. Cut-off values, sensitivity, and specificity of the indirect ELISA were determined in correlation to the SNT results using a receiver operating characteristic analysis (ROC analysis) with regard to the criterion “maximization of sensitivity and specificity”. Calculations were performed using the R program and the R package “OptimalCutpoints” [14,15].

### 2.7. Statistical Analysis

The estimated prevalences and 95% confidence intervals (95% CIs) were calculated using the Epitools calculation tool (https://epitools.ausvet.com.au/ciproportion), accessed on 14 January 2021.

## 3. Results

A total of 325 ruminants (121 sheep, 60 goats, and 144 cattle) from Saxony-Anhalt in Eastern Germany were tested by qRT-PCR for BATV genomes. BATV-specific RNA was, however, not detected. The serological analysis was performed by a virus-specific SNT and additionally by an indirect ELISA. Four out of the 144 blood samples from the cattle showed cytotoxic effects on the cells and were therefore not included in the data evaluation. Of the remaining 140 cattle samples, 58 specimens revealed neutralizing antibodies (seroprevalence of 41.4%) (Table 1).

**Figure 1:**
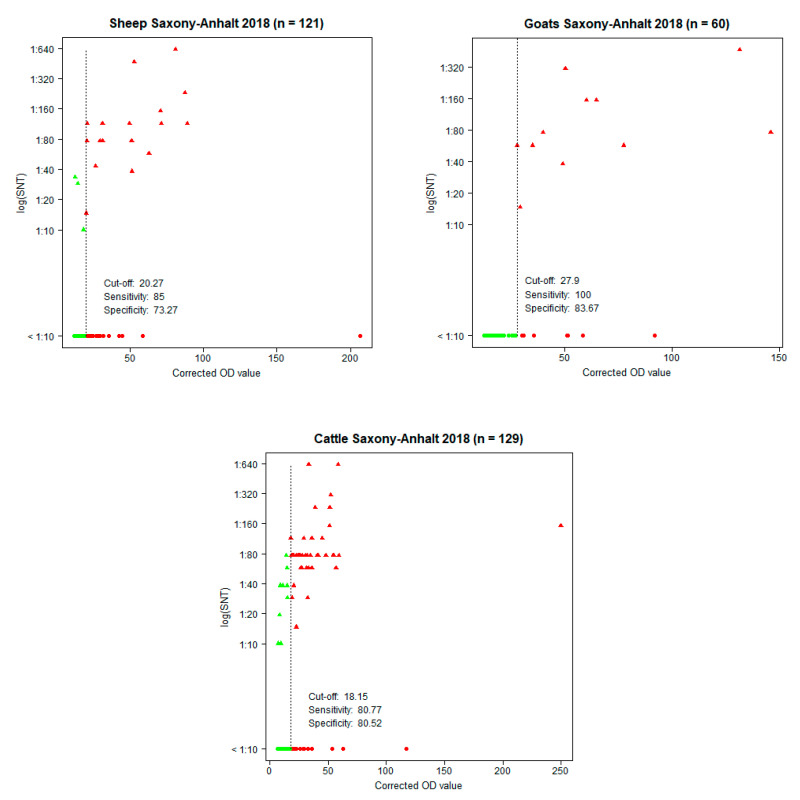
Corrected optical density (OD) values of the ELISA in relation to the neutralization titers (log (SNT)) of the SNT showing the cut-off, sensitivity, and specificity for each species. Green dots: SNT- and ELISA-negative samples. Green triangles: SNT-positive, but ELISA-negative samples. Red triangles: SNT- and ELISA-positive samples. Red dots: SNT-negative, but ELISA-positive samples.

Thereby, in almost every investigated flock, antibody-positive cattle were detected, whereby the highest number of positive animals was found in three flocks (Zehrental, Schönhausen, and Müggenbusch) (Supplemental Table S1). The prevalences in the sheep and goats were in a similar range (16.5% and 18.3%, respectively). Thereby, especially in two sheep flocks (Haldensleben and Osternienburger Land) and exclusively in one goat flock (Schleckweda), positive animals were detected. An indirect ELISA was implemented and resulting OD values were compared with the results from the SNT, which were used as a reference and as a gold standard. Eleven of 140 cattle sera were not tested in the ELISA, due to low sample volume. The highest correlation was achieved with a cut-off at a PP value of 18.2 for the cattle, yielding a specificity of 80.5% and a sensitivity of 80.8%, for the sheep a cut-off at a PP value of 20.3, yielding a specificity of 73.3% and a sensitivity of 85.0%, and for the goats a cut-off at a PP value of 27.9%, yielding a specificity of 83.7% and a sensitivity of 100% (Figure 1). Both assays classified 69 of the 310 analyzed sera as positive (Table 2). In addition, 51 sera were ELISA positive, but negative in the SNT. Interestingly, 37 of these 51 samples had low PP values of ≤36%. By contrast, only 13 sera were positive in the SNT, but were found negative by the ELISA.

## 4. Discussion

The presence of BATV antibodies in the sheep, goat, and cattle samples collected in the time period of 2013 to 2016 in Eastern Germany was demonstrated recently. High BATV seroprevalences were found in Saxony-Anhalt (44.7%, 38.8%, and 36.4%, respectively) [9]. Thus, it was assumed that ruminants are susceptible to BATV and that the virus is endemically circulating in Eastern Germany [9]. This conclusion was confirmed by the subsequent detection of viral RNA in anopheline and culicine mosquitoes in the very same region [8]. In Southwestern Germany, BATV was detected even earlier in mosquitoes [7]. However, a subsequent seroprevalence study in ruminants only revealed three antibody-positive cattle out of 548 tested animals [16], indicating regional differences in virus prevalence within Germany.

**Figure 2:**
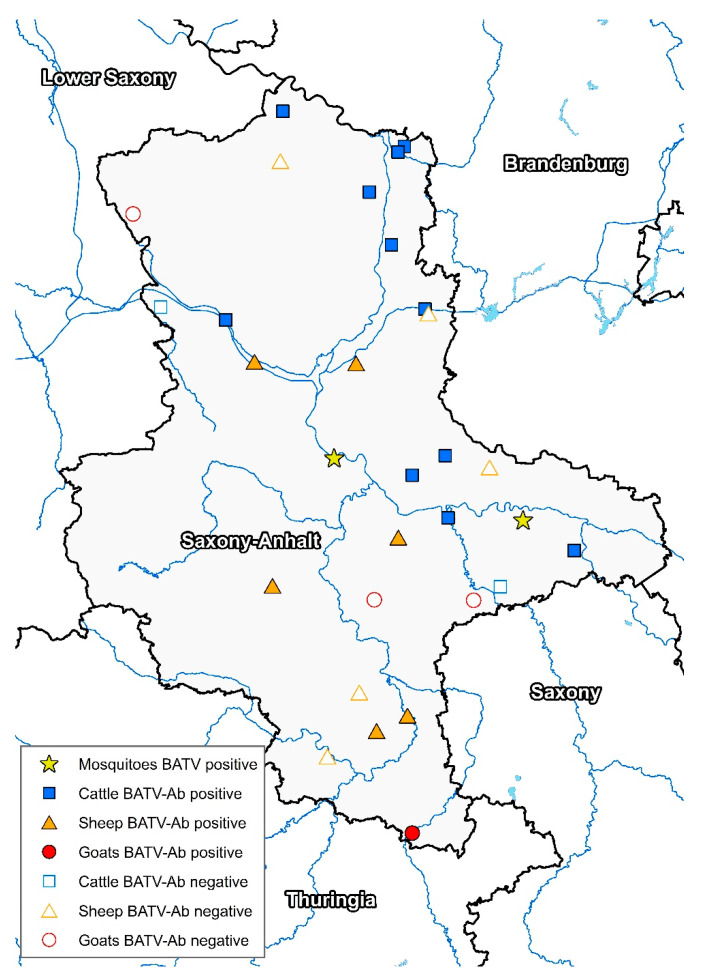
Geographical distribution of the investigated ruminant flocks in Saxony-Anhalt in combination with the serological results (BATV-Ab = BATV antibodies) and the sites with BATV-positive mosquitoes (virus or viral RNA).

To further monitor BATV circulation in the most affected region in Eastern Germany, the present follow-up study conducted a molecular and serological analysis in 18 ruminant flocks in Saxony-Anhalt (Figure 2). As viral RNA was not detected, the animals were probably not viremic during the sampling period.

To date, serological investigations for BATV have been conducted by hemagglutination inhibition tests (HI), SNTs, plaque reduction neutralization tests (PRNTs), and by immunofluorescence assays (IFAs). As these sensitive tests depend on the prior cultivation of live virus, they require higher biosafety standards [9,16,17,18]. Therefore, we implemented an indirect ELISA based on the partial recombinant BATV glycoprotein Gc for BATV Gc-specific antibodies. ELISAs based on recombinant proteins are fast and reliable, permitting the testing of larger sample sizes for monitoring purposes. Compared to the SNT, the ELISA established here exhibits suitable sensitivity and specificity levels for initial serology. Hence, we recommend to screen samples with the ELISA first, and to verify the positive samples with the SNT in a second step. A subsequent confirmation via an SNT is especially important for samples with a low OD value near the cut-off value. In future, efforts could be made to increase the sensitivity and specificity of the ELISA and to further reduce the necessity of an SNT.

Antibodies against BATV were found in all three species. The sheep and goats showed moderate BATV prevalences (16.5% and 18.3%, respectively) whereas the cattle had the highest antibody incidence (41.4%). In 2018, we observed a significantly lower seroprevalence than in the earlier study from Ziegler et al. [9]. Obviously, this could also be due to a sampling artefact as different sets of animals were tested. Interestingly, when ELISA-positive samples were tested by the SNT, the more recent samples also had lower antibody titers: in 2018, only a few samples showed a titer of 1:640 and most of the titers did not exceed 1:120, whereas in 2013 to 2016, antibody titers were up to 1:2560 [9].

In other European countries (Finland, Austria, Slovakia, Portugal, Romania, and former Yugoslavia) antibody titers detected by HI ranged from 1% to 46% in cattle and sheep [17]. A recent study in northern Italy found nine antibody-positive cattle out of 128 tested animals, corresponding to a prevalence of 7% [18]. In humans, the prevalence of HI antibodies was generally very low (<1%) in Sweden, Finland, Germany, Austria, and former Yugoslavia, but reached 32% in southern Slovakia [17]. The previously published data along with the results of our study show that BATV is circulating widely in Europe to a low to moderate extent.

Besides mild flu-like illnesses in humans [6], infections with BATV appear not to cause clinical signs in the majority of the vertebrate hosts, and, therefore, may only pose a minor threat to human and veterinary public health. However, a German harbor seal with fatal encephalitis was found to be naturally infected with BATV [5]. Furthermore, infections with the closely related Cache Valley virus in America are associated with stillbirth and congenital abnormalities in ruminants [19] and encephalitis in humans [20].

BATV contributed a parental segment donor in a natural reassortment event [21]. Coinfection of BATV and Bunyamwera virus (BUNV) resulted in the generation of a virulent progeny, the so-called Ngari virus (NRIV), which caused two major hemorrhagic fever outbreaks in humans in Africa [1]. Apparently, this reassortment led to an increase in pathogenicity. However, infection studies with NRIV and its parental viruses in ruminants are scarce or even missing [1]. The reassortant NRIV possesses the S and L segments from BUNV combined with the M segment from BATV. It probably evolved in coinfected mosquitoes [1], as mosquitoes can feed on different vertebrates possibly infected with distinct viruses [3]. M segment gene products (Gc, Gn and NSm) have a major influence on the vector competence (ability to efficiently transmit a virus) [3]. Thus, the risk of NRIV transmission by autochthonous mosquitoes in Europe, which are demonstrated to be competent for BATV, should not be neglected and could be addressed by vector competence studies.

## Figures and Tables

**Table 1:** Serological analysis of the ruminant blood samples for Batai virus (BATV) with serum neutralization test (SNT).

Species	Flocks	Tested Animals	Positive Flocks	Positive Animals	Prevalence (%)	95% CI
Sheep	11	121	6	20	16.5	11.0–24.2
Goats	4	60	1	11	18.3	10.6–29.9
Cattle	13	140	11	58	41.4	33.6–49.7
Total	18	321	89	89	27.7	23.1–32.9

**Table 2:** Summary of SNT and indirect ELISA results for each species: (a) sheep, (b) goats, (c) cattle, and (d) in total.

**(a) Sheep**			
	ELISA positive	ELISA negative	Total
SNT positive	17	3	20
SNT negative	27	74	101
Total	44	77	121
**(b) Goats**			
	ELISA positive	ELISA negative	Total
SNT positive	11	0	11
SNT negative	8	41	49
total	19	41	60
			
**(c) Cattle**			
	ELISA positive	ELISA negative	Total
SNT positive	41	10	51
SNT negative	16	62	78
Total	57	72	129
			
**(d) All species**			
	ELISA positive	ELISA negative	Total
SNT positive	69	13	82
SNT negative	51	177	228
Total	120	190	310

## Data Availability

Data generated or analyzed during this study are included in the published article and in the Supplementary Materials.

## Supplementary Materials

The following are available online at https://www.mdpi.com/1999-4915/13/3/370/s1, Table S1: Percent positivity among individual flocks of each species: (a) sheep, (b) goats, and (c) cattle.
