# Supplementary material for: Serological and Molecular Investigation of Batai Virus Infections in Ruminants from the State of Saxony-Anhalt, Germany, 2018
Source: Viruses. 2021 Feb 26;13(3):370. doi: 10.3390/v13030370 (PMC7996813; doi:10.3390/v13030370)
Supplement: Supplementary file 1 [file viruses-13-00370-s001.pdf]

Supplementary Table S1:

Percent positivity among individual flocks of each species a) sheep, b) goats, and c) cattle.

a) Sheep

Flocks/regions	Total	Positive animals	Percent Positivity (%)
Laucha/U.	10	0	0
Haldensleben	11	8	72.7
Grochewitz	20	0	0
Genthin	10	0	0
Kossebau	10	0	0
Bad Lauchstädt	10	0	0
Arnstein	10	1	10
Braunsbedra	10	2	20
Leuna	10	1	10
Osternienburger Land	10	7	70
Burg	10	1	10
Total	121	20	16.5

b) Goats

Flocks/regions	Total	Positive animals	Percent Positivity (%)
Löbejün	20	0	0
Schleckweda	20	11	55
Bitterfeld Wolfen	10	0	0
Jübar	10	0	0
Total	60	11	18.3

c) Cattle

Flocks/regions	Total	Positive animals	Percent Positivity (%)
Hohenberg	10	2	20
Zehrental	6	6	100
Schönhausen	10	9	90
Zerbst/Anhalt	10	3	30
Muldestausee	12	0	0
Müggenbusch	12	12	100
Havelberg	12	5	41.7
Calvörde	12	4	33.3
Dessau-Roßlau	11	6	54.5
Kleinzerbst	12	3	25
Mühro	12	7	58.3
Weddendorf	9	0	0
Genthin	12	1	8.3
Total	140	58	41.4
